# Association of systemic inflammation and body mass index with survival in patients with resectable gastric or gastroesophageal junction adenocarcinomas

**DOI:** 10.20892/j.issn.2095-3941.2020.0246

**Published:** 2021-02-15

**Authors:** Xianchun Gao, Yanan Pan, Weili Han, Caie Hu, Chenchen Wang, Ling Chen, Yong Guo, Yupeng Shi, Yan Pan, Huahong Xie, Liping Yao, Jianjun Yang, Jianyong Zheng, Xiaohua Li, Xiaonan Liu, Liu Hong, Jipeng Li, Mengbin Li, Gang Ji, Zengshan Li, Jielai Xia, Qingchuan Zhao, Daiming Fan, Kaichun Wu, Yongzhan Nie

**Affiliations:** 1 State Key Laboratory of Cancer Biology and National Clinical Research Center for Digestive Diseases, Xijing Hospital, Air Force Medical University of PLA, Xi’an 710032, China; 2 School of Life Science, Northwest University, Xi’an 710069, China; 3 Department of Gastroenterology, The First Affiliated Hospital of Xi’an Medical University, Xi’an 710021, China; 4 State Key Laboratory of Cancer Biology, Department of Pathology, Xijing Hospital and School of Basic Medicine, Air Force Medical University of PLA, Xi’an 710032, China; 5 Department of Medical Statistics, School of Preventive Medicine, Air Force Medical University of PLA, Xi’an 710032, China

**Keywords:** Gastric cancer, neutrophil-to-lymphocyte ratio, body mass index, prognosis, systemic inflammation index

## Abstract

**Objective:**

The systemic inflammation index and body mass index (BMI) are easily accessible markers that can predict mortality. However, the prognostic value of the combined use of these two markers remains unclear. The goal of this study was therefore to evaluate the association of these markers with outcomes based on a large cohort of patients with gastric cancer.

**Methods:**

A total of 2,542 consecutive patients undergoing radical surgery for gastric or gastroesophageal junction adenocarcinoma between 2009 and 2014 were included. Systemic inflammation was quantified by the preoperative neutrophil-to-lymphocyte ratio (NLR). High systemic inflammation was defined as NLR ≥ 3, and underweight was defined as BMI < 18.5 kg/m^2^.

**Results:**

Among 2,542 patients, NLR ≥ 3 and underweight were common [627 (25%) and 349 (14%), respectively]. In the entire cohort, NLR ≥ 3 or underweight independently predicted overall survival (OS) [hazard ratio (HR): 1.236, 95% confidence interval (95% CI): 1.069–1.430; and HR: 1.600, 95% CI: 1.350–1.897, respectively] and recurrence-free survival (RFS) (HR: 1.230, 95% CI: 1.054–1.434; and HR: 1.658, 95% CI: 1.389–1.979, respectively). Patients with both NLR ≥ 3 and underweight (*vs.* neither) had much worse OS (HR: 2.445, 95% CI: 1.853–3.225) and RFS (HR: 2.405, 95% CI: 1.802–3.209). Furthermore, we observed similar results in subgroup analyses according to pathological stage, age, and postoperative chemotherapy.

**Conclusions:**

Our results showed that preoperative elevated NLR and decreased BMI had a significant negative effect on survival. Underweight combined with severe inflammation could enhance prognostication. Taking active therapeutic measures to reduce inflammation and increase nutrition may help improve outcomes.

## Introduction

Gastric cancer is globally the third leading cause of cancer-related death among males and the fifth leading cause of cancer-related death among females^1^. The incidence rates vary widely across regions and are highest in Eastern Asia^1^. Even in patients with resectable gastric or gastroesophageal junction adenocarcinomas, the prognoses remain dismal. Compared with surgery alone, several effective approaches improve survival, including perioperative chemotherapy or chemoradiation^2–4^. However, in most cases, the prognoses of patients with gastric or gastroesophageal junction adenocarcinomas rely on histopathological tumor staging according to the American Joint Committee on Cancer/Union for International Cancer Control (UICC) tumor-node-metastasis (TNM) classification system. This risk stratification system provides useful but imprecise prognostic information. In clinical practice, the prognosis is clearly different even in patients with the same pathological classification. Systemic factors, including inflammation, metabolism, nutrition, and immunity, are important prognostic factors. Accurate staging is therefore essential for classifying high risk patients to provide more rational management and individualized treatment decisions.

The associations between the occurrence and progression of tumors and inflammation have been recognized for many years^5,6^. Systemic inflammatory markers, such as acute phase proteins (C-reactive protein^7^ or interleukin-6^8^) and markers based on routine blood tests [neutrophil-to-lymphocyte ratio (NLR) or platelet-to-lymphocyte ratio]^9^, have been studied as prognostic and predictive factors, receiving increasing attention in gastric cancer. NLR has been used as an easily acquired marker for the systemic inflammatory response. Elevated NLR is associated with high serum levels of various cytokines^10^, and increased NLR is significantly associated with poorer response to treatment and worse survival^11,12^. However, little is known about the influence of NLR on survival in subgroups of patients stratified by age, cancer stage, and adjuvant chemotherapy.

In addition to the systemic inflammatory response, nutritional status is another important factor affecting oncological outcomes across cancer types^13–15^. One useful method for assessing nutritional status is body mass index (BMI). There is increasing evidence that low BMI before surgery or decreased BMI during chemotherapy is associated with poor survival in gastric cancer patients^16–18^. The potential correlation between high BMI and better therapeutic responses to targeted therapy or immune therapy has also been observed in patients with metastatic melanoma^19^. The incidence rates of gastric cancer are the highest in Eastern Asian countries, but the dietary habits, living environment, and underweight rates are different among countries. Currently, large population studies on the relationship between BMI and the prognosis of gastric cancer in Chinese patients are rare.

NLR and BMI as independent prognostic indicators are attracting increasing attention, but the relationship between these factors and their combined associations with prognoses are not well studied. The co-occurrence of high inflammation and underweight associated with a much poorer prognosis was observed in patients with metastatic renal cell carcinoma^20^. To the best of our knowledge, no prior study has evaluated the combined associations of systemic inflammatory markers and BMI measured prior to surgery with gastric cancer prognoses. Accordingly, we examined the independent and combined associations of NLR and BMI at diagnosis with postoperative survival in patients with adenocarcinomas of the stomach or gastroesophageal junction. Subsequently, we examined whether the associations were consistent in subgroup analyses by age, pathological TNM stage, and chemotherapy.

## Materials and methods

### Study population

Our study population included consecutive patients diagnosed with stage I–III gastric or gastroesophageal junction adenocarcinomas (*n* = 2,753) at the Xijing Hospital between 2009 and 2014. Patients with prior gastric surgery, multiple primary cancers in other organs, preoperative chemotherapy, no blood test results, or missing weight and height at diagnosis were excluded from the analysis. Finally, a total of 2,542 remaining consecutive patients treated with radical surgery without residual malignant disease were enrolled in this study. The study was approved by the Ethical Committee of Xijing Hospital (Approval No. KY20192088-F-1). The requirement for informed consent was exempted for this retrospective analysis of a prospective database.

### NLR and BMI

As the preoperative marker of systemic inflammation used in this study, NLR was extracted in a retrospective manner from electronic medical records as a part of routine blood tests at diagnosis prior to surgery. The patients were divided into “low” (< 3) and “high” (≥ 3) inflammation groups based on the most common cut-off value of 3. This cut-off value was determined based on previous studies^11,21,22^. BMI was calculated as the patient’s weight (in kg) on the admission day for gastrectomy divided by the square of the height (in m). The patients were then divided into 2 groups according to the World Health Organization BMI classification for Asian populations: underweight, BMI < 18.5 kg/m^2^; and normal weight or overweight, BMI ≥ 18.5 kg/m^2,23^.

### Other covariates and follow-up

We reviewed the electronic medical records for data on clinicopathological diagnosis, blood biochemistry, tumor markers, surgery, surgical complications (defined as grade II or higher of the Clavien-Dindo classification^24^), chemotherapy, past medical history, and demographic information. The patients were followed prospectively in 6 to 12 month intervals, and the results of laboratory tests, computed tomography scans, and gastroscopy were recorded. The last follow-up date was July 8, 2019.

### Statistical analysis

Differences in clinicopathological characteristics at baseline were described according to NLR and BMI status and were compared with one-way analysis of variance for continuous variables or Pearson’s χ^2^ test for categorical variables. Pearson’s correlation coefficient analysis was used to assess the correlation between NLR and BMI. Overall survival (OS) was defined as the time from the date of primary surgery to patient death from any cause. Recurrence-free survival (RFS) was defined as the time from the date of primary surgery until the first evidence of recurrence. Survival curves were derived using the Kaplan-Meier method, and the log-rank test was used to evaluate differences between survival curves. Next, we examined NLR ≥ 3 and being underweight as independent predictors of survival in multivariable Cox proportional hazards models. The models were adjusted for the age category, sex, adjuvant chemotherapy, primary tumor location, histopathological differentiation, number of lymph nodes dissected, TNM stage, family history of gastric cancer, Charlson comorbidity score, current smoker status, alcohol use, anemia, hypoalbuminemia, type of gastrectomy, and surgical complications. We also performed subgroup analyses based on the TNM stage, age (< 55 *vs.* ≥ 55 to < 65 *vs.* ≥ 65), and treatment arm. When subgroup analyses were performed, age category, sex, adjuvant chemotherapy, primary tumor location, histopathological differentiation, number of lymph nodes dissected, TNM stage, family history of gastric cancer, Charlson comorbidity score, smoker status, alcohol use, anemia, hypoalbuminemia, type of gastrectomy, and surgical complications were adjusted unless stratified by those variables. A *P* value of < 0.05 was regarded as significant. All analyses were performed using SPSS statistical software for Windows, version 22.0 (SPSS, Armonk, NY, USA).

## Results

### Associations between NLR and BMI and patient baseline variables

A total of 2,542 patients (1,962 males and 580 females) were investigated in this study, and the median follow-up was 6.3 years. The median age was 58 years, with a range of 21–90 years. In 627 patients (25%), the NLR was above the cut-off value of 3. The median NLR was 2.10 (interquartile range: 1.54–2.97). The minority of patients were underweight (14%), and the median BMI was 22.0 kg/m^2^ (interquartile range: 19.8–24.2 kg/m^2^). The prevalence of neither NLR ≥ 3 nor underweight and both NLR ≥ 3 and underweight was 65.5% (*n* = 1,664) and 3.9% (*n* = 98), respectively. Pearson’s correlation analysis showed no significant correlation between the at-diagnosis NLR and BMI (Pearson’s coefficient: -0.030, *P* = 0.130). Furthermore, among advanced patients (stages II and III), there was still no significant correlation between NLR and BMI (Pearson’s coefficient: -0.022, *P* = 0.345).

We then assessed the associations between NLR and BMI and the clinicopathological characteristics of patients (**Table 1**). Significant differences were found among the 4 groups in age, sex, Charlson comorbidity score, current smoker status, primary tumor location, type of gastrectomy, pathological TNM stage, tumor stage, lymph node stage, number of lymph nodes dissected, preoperative serum albumin, and anemia. Elevated NLR or decreased BMI was strongly associated with many variables previously shown to be associated with a poor prognosis. Patients with both NLR ≥ 3 and underweight (*vs.* neither) were older; more likely to be female; to be nonsmokers; to have antrum cancer; to receive distal gastrectomy; to have advanced tumor stage, lymph node stage, and pathological TNM stage; to have over 16 lymph nodes dissected; and to have hypoalbuminemia and anemia. As shown in **Supplementary Figure S1**, the proportion of both NLR ≥ 3 and underweight increased proportionally with increasing pathological stage. However, in terms of surgical complications, postoperative 30-day mortality, and adjuvant chemotherapy, there were no significant differences among the 4 groups. The effects of age on NLR and BMI were relatively complicated. **Supplementary Figure S2** shows the age distribution according to NLR and BMI. The proportions of patients with either high NLR or underweight were prone to be low in middle-aged patients. With increased age, the proportions of either high NLR or underweight individuals tended to decrease first and then increase with age.

**Table 1:** Baseline characteristics according to the neutrophil-to-lymphocyte ratio and body mass index at diagnosis

Characteristics	All patients (*n* = 2,542)	Neither^a^ (*n* = 1,664)	Only NLR ≥ 3 (*n* = 529)	Only BMI < 18.5 (*n* = 251)	Both^b^ (*n* = 98)	*P*
Age (years)						< 0.001
≥ 65	696 (27%)	408 (25%)	166 (31%)	84 (34%)	38 (39%)	
55 to < 65	930 (37%)	603 (36%)	200 (38%)	94 (38%)	33 (34%)	
< 55	916 (36%)	653 (39%)	163 (31%)	73 (29%)	27 (28%)	
Gender						< 0.001
Male	1962 (77%)	1313 (79%)	421 (80%)	165 (66%)	63 (64%)	
Female	580 (23%)	351 (21%)	108 (20%)	86 (34%)	35 (36%)	
CCS, mean (SD)	0.16 (0.44)	0.15 (0.42)	0.22 (0.52)	0.13 (0.40)	0.14 (0.41)	0.012
Smoke						0.006
No	1633 (64%)	1036 (62%)	348 (66%)	184 (73%)	65 (66%)	
Yes	909 (36%)	628 (38%)	181 (34%)	67 (27%)	33 (34%)	
Alcohol						0.220
No	2228 (88%)	1448 (87%)	463 (88%)	230 (92%)	87 (89%)	
Yes	314 (12%)	216 (13%)	66 (13%)	21 (8%)	11 (11%)	
Family history of gastric cancer						0.685
No	2383 (94%)	1557 (94%)	494 (93%)	238 (95%)	94 (96%)	
Yes	159 (6%)	107 (6%)	35 (7%)	13 (5%)	4 (4%)	
Primary tumor location						0.002
Proximal	776 (31%)	505 (30%)	192 (36%)	60 (24%)	19 (19%)	
Body	610 (24%)	395 (24%)	126 (24%)	64 (26%)	25 (26%)	
Antrum	1156 (46%)	764 (46%)	211 (40%)	127 (51%)	54 (55%)	
Type of gastrectomy						< 0.001
Proximal	262 (10%)	185 (11%)	53 (10%)	19 (8%)	5 (5%)	
Total	1190 (47%)	744 (45%)	291 (55%)	109 (43%)	46 (47%)	
Distal	1090 (43%)	735 (44%)	185 (35%)	123 (49%)	47 (48%)	
Pathologic TNM stage^c^						< 0.001
Stage I	660 (26%)	476 (29%)	107 (20%)	66 (26%)	11 (11%)	
Stage II	651 (26%)	429 (26%)	136 (26%)	56 (22%)	30 (31%)	
Stage III	1231 (48%)	759 (46%)	286 (54%)	129 (51%)	57 (58%)	
T stage						< 0.001
T1	494 (19%)	365 (22%)	80 (15%)	42 (17%)	7 (7%)	
T2	396 (16%)	270 (16%)	67 (13%)	45 (18%)	14 (14%)	
T3	467 (18%)	299 (18%)	112 (21%)	33 (13%)	23 (24%)	
T4	1185 (47%)	730 (44%)	270 (51%)	131 (52%)	54 (55%)	
N stage						0.012
N0	981 (39%)	670 (40%)	184 (35%)	101 (40%)	26 (27%)	
N1	472 (19%)	315 (19%)	99 (19%)	39 (16%)	19 (19%)	
N2	450 (18%)	297 (18%)	94 (18%)	43 (17%)	16 (16%)	
N3	639 (25%)	382 (23%)	152 (29%)	68 (27%)	37 (38%)	
No. of lymph nodes dissected						0.014
≥16	2152 (85%)	1383 (83%)	458 (87%)	221 (88%)	90 (92%)	
<16	390 (15%)	281 (17%)	71 (13%)	30 (12%)	8 (8%)	
Histopathological differentiation						0.153
High/middle	989 (39%)	666 (40%)	199 (38%)	92 (37%)	32 (33%)	
Poor	1443 (57%)	934 (56%)	305 (58%)	141 (56%)	63 (64%)	
Signet ring cell or Mucinous	110 (4%)	64 (4%)	25 (5%)	18 (7%)	3 (3%)	
Serum albumin (g/l)						< 0.001
≥40	1878 (74%)	1293 (78%)	376 (71%)	157 (63%)	52 (53%)	
<40	664 (26%)	371 (22%)	153 (29%)	94 (38%)	46 (47%)	
Anaemia						< 0.001
No	1883 (74%)	1321 (79%)	335 (63%)	174 (69%)	53 (54%)	
Yes	659 (26%)	343 (21%)	194 (37%)	77 (31%)	45 (46%)	
Surgical complications^d^						0.913
No	2090 (82%)	1369 (82%)	437 (83%)	206 (82%)	78 (80%)	
Yes	452 (18%)	295 (18%)	92 (17%)	45 (18%)	20 (20%)	
30-day mortality						0.076
No	2521 (99.2%)	1654 (99.4%)	521 (98.5%)	250 (99.6%)	96 (98.0%)	
Yes	21 (0.8%)	10 (0.6%)	8 (1.5%)	1 (0.4%)	2 (2.0%)	
Treatment arm						0.193
Surgery only	853 (34%)	554 (33%)	169 (32%)	99 (39%)	31 (32%)	
Surgery + chemotherapy	1689 (66%)	1110 (67%)	360 (68%)	152 (61%)	67 (68%)	

^a^Indicating NLR < 3 and BMI ≥ 18.5 kg/m^2^; ^b^Indicating NLR ≥ 3 and underweight (BMI < 18.5 kg/m^2^); ^c^8th UICC staging system; ^d^Defined as grade II or higher using the Clavien-Dindo classification. NLR, neutrophil-to-lymphocyte ratio; BMI, body mass index; CCS, Charlson comorbidity score; SD, standard deviation; TNM, tumor-node-metastasis; T stage, tumor stage; N stage, node stage.

### Survival analysis

**Figure 1:**
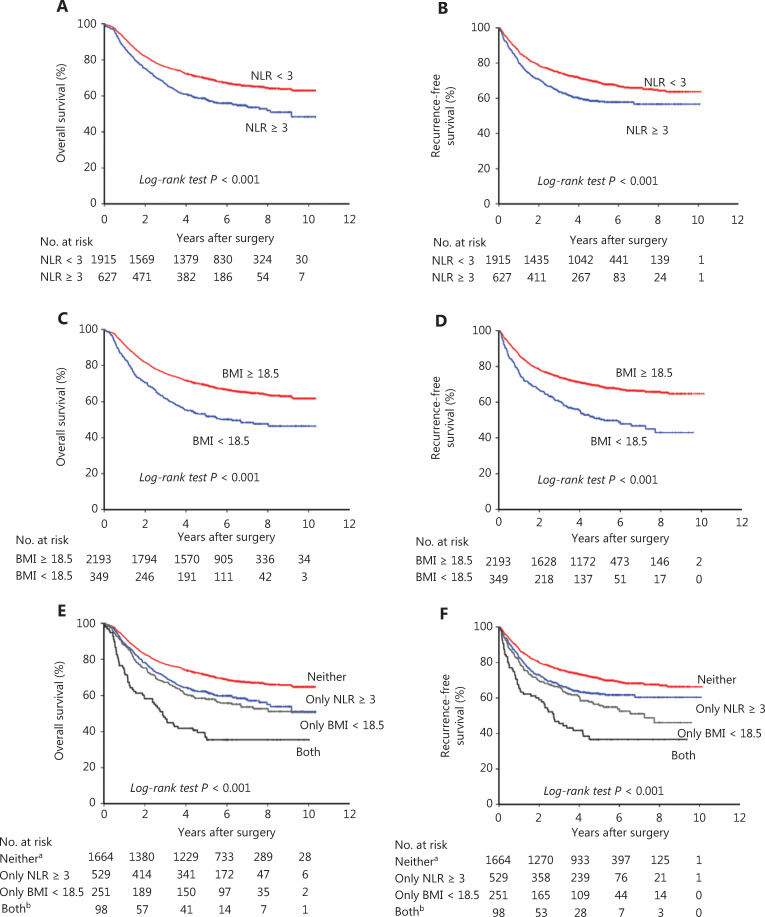
Kaplan-Meier survival curves according to the neutrophil-to-lymphocyte ratio (NLR) and body mass index (BMI) at diagnosis in resectable gastric cancer patients. (A) Overall survival according to NLR in 2,542 patients. (B) Recurrence-free survival according to NLR in 2,542 patients. (C) Overall survival according to BMI in 2,542 patients. (D) Recurrence-free survival according to BMI in 2,542 patients. (E) Overall survival according to NLR and BMI in 2,542 patients. (F) Recurrence-free survival according to NLR and BMI in 2,542 patients. ^a^Neither, indicating NLR < 3 and BMI ≥ 18.5 kg/m^2^; ^b^both, indicating NLR ≥ 3 and underweight (BMI < 18.5 kg/m^2^).

Of the 2,542 patients, 918 (36%) died. As observed in the Kaplan-Meier curves (**Figure 1**), patients with high NLR or underweight had a worse prognosis. When the patients were stratified into two categories according to NLR, those with NLR ≥ 3 were associated with a worse prognosis, with lower 5-year OS (58% *vs.* 70%, *P* < 0.001, **Figure 1A**) and RFS rates (58% *vs.* 69%, *P* < 0.001, **Figure 1B**). When the patients were stratified into two categories according to BMI, those who were underweight were associated with a worse prognosis, with a lower 5-year OS (52% *vs.* 69%, *P* < 0.001, **Figure 1C**) and RFS rates (50% *vs.* 69%, *P* < 0.001, **Figure 1D**). When the patients were stratified into 4 categories according to NLR and BMI, patients with both NLR ≥ 3 and underweight had the worst prognosis, whereas patients with NLR < 3 who were not underweight survived the longest (5-year OS: 71% *vs.* 37%, *P* < 0.001, **Figure 1E**; 5-year RFS: 71% *vs.* 36%, *P* < 0.001, **Figure 1F**); the survival rates of patients with only NLR ≥ 3 or underweight were between the above 2 (5-year OS: 62% *vs.* 58%, *P* = 0.354, **Figure 1E**; 5-year RFS: 62% *vs.* 55%, *P* = 0.095, **Figure 1F**).

The mutually adjusted effects of NLR and BMI on OS and RFS were detailed in **Table 2**. In the multivariable model, age, sex, adjuvant chemotherapy, primary tumor location, histopathological differentiation, number of lymph nodes dissected, TNM stage, family history of gastric cancer, Charlson comorbidity score, current smoker status, alcohol use, anemia, hypoalbuminemia, type of gastrectomy, and surgical complications were adjusted as covariates. Elevated NLR and underweight were independently associated with a worse OS [hazard ratio (HR): 1.236, 95% confidence interval (95% CI): 1.069–1.430; and HR: 1.600, 95% CI: 1.350–1.897, respectively] and RFS (HR: 1.230, 95% CI: 1.054–1.434; and HR: 1.658, 95% CI: 1.389–1.979, respectively). In addition, patients with both elevated NLR and underweight were estimated to have the worst OS (adjusted HR for both elevated NLR and underweight *vs.* neither, 2.445, 95% CI: 1.853–3.225) and RFS (adjusted HR for both elevated NLR and underweight *vs.* neither 2.405; 95% CI: 1.802–3.209) among the 4 categories.

**Table 2:** Mutually adjusted effects of neutrophil-to-lymphocyte ratio and body mass index and survival in 2,542 patients.

	Patients No.	Events No.	Univariable model HR (95% CI)	*P* value	Multivariable model 1^a^ HR (95% CI)	*P*	Multivariable model 2^b^ HR (95% CI)	*P*
Overall survival								
NLR				< 0.001		< 0.001		0.004
≥3	627	279	1.497 (1.300–1.724)		1.280 (1.111–1.475)		1.236 (1.069–1.430)	
<3	1915	639	1		1		1	
BMI (kg/m^2^)				< 0.001		< 0.001		< 0.001
<18.5	349	176	1.722 (1.461–2.030)		1.607 (1.360–1.899)		1.600 (1.350–1.897)	
≥18.5	2193	742	1		1		1	
NLR and BMI (kg/m^2^)^c^				< 0.001		< 0.001		< 0.001
Both^d^	98	62	2.882 (2.213–3.752)		2.469 (1.891–3.225)		2.445 (1.853–3.225)	
Neither^e^	1664	525	1		1		1	
Recurrence-free survival								
NLR				< 0.001		< 0.001		0.008
≥3	627	246	1.447 (1.246–1.679)		1.257 (1.082–1.460)		1.230 (1.054–1.434)	
<3	1915	584	1		1		1	
BMI (kg/m^2^)				< 0.001		< 0.001		< 0.001
<18.5	349	165	1.804 (1.521–2.140)		1.672 (1.407–1.988)		1.658 (1.389–1.979)	
≥18.5	2193	665	1		1		1	
NLR and BMI (kg/m^2^)^c^				< 0.001		< 0.001		< 0.001
Both^d^	98	57	2.821 (2.142–3.715)		2.404 (1.818–3.177)		2.405 (1.802–3.209)	
Neither^e^	1664	476	1		1		1	

^a^Age, sex, adjuvant chemotherapy, and TNM stage were adjusted in multivariable model 1 for overall survival and recurrence-free survival; ^b^Age, sex, adjuvant chemotherapy, primary tumor location, histopathological differentiation, number of lymph nodes dissected, TNM stage, family history of gastric cancer, Charlson comorbidity score, smoke, alcohol, anemia, hypoalbuminemia, type of gastrectomy and surgical complications were adjusted for a multivariable model 2 for overall survival and recurrence-free survival; ^c^Patients were divided into 4 groups according to NLR and BMI: neither NLR ≥ 3 nor underweight (BMI < 18.5 kg/m^2^); NLR ≥ 3 only; underweight only; both NLR ≥ 3 and underweight; ^d^Indicating NLR ≥ 3 and underweight (BMI < 18.5 kg/m^2^); ^e^indicating NLR < 3 and BMI ≥ 18.5 kg/m^2^. NLR, neutrophil-to-lymphocyte ratio; BMI, body mass index; HR, hazard ratio; CI, confidence intervals; TNM, tumor-node-metastasis.

### Subgroup analyses according to age, pathological stage, and adjuvant chemotherapy

As NLR and BMI were closely associated with other factors affecting survival, including age, pathological TNM stage, and adjuvant chemotherapy, we further investigated their prognostic values in subgroups (**Table 3**).

**Table 3:** Subgroup analysis for neutrophil-to-lymphocyte ratio and body mass index at diagnosis according to pathologic stage, age and adjuvant chemotherapy.

Stratification Variable	Overall Survival				Recurrence–free survival			
	Univariable model HR (95% CI)	*P* value	Multivariable model^a^ HR (95% CI)	*P* value	Univariable model HR (95% CI)	*P* value	Multivariable model^a^ HR (95% CI)	*P* value
NLR^b^								
Pathologic TNM stage								
Stage I	1.666 (0.931–2.984)	0.086	1.705 (0.913–3.185)	0.094	0.817 (0.367–1.816)	0.619	0.985 (0.451–2.154)	0.970
Stage II	1.555 (1.124–2.150)	0.008	1.308 (0.929–1.842)	0.123	1.593 (1.128–2.249)	0.008	1.410 (0.980–2.031)	0.064
Stage III	1.246 (1.059–1.466)	0.008	1.186 (1.003–1.404)	0.047	1.245 (1.050–1.476)	0.012	1.190 (0.997–1.420)	0.054
Age at diagnosis, years								
≥65	1.359 (1.075–1.719)	0.010	1.189 (0.927–1.525)	0.174	1.262 (0.972–1.640)	0.081	1.184 (0.898–1.560)	0.232
55 to <65	1.792 (1.428–2.248)	< 0.001	1.485 (1.175–1.876)	0.001	1.695 (1.337–2.148)	< 0.001	1.422 (1.113–1.816)	0.005
<55	1.175 (0.883–1.563)	0.269	1.036 (0.773–1.389)	0.812	1.251 (0.939–1.668)	0.126	1.113 (0.828–1.496)	0.477
Treatment arm								
Surgery only	1.619 (1.273–2.060)	< 0.001	1.361 (1.058–1.750)	0.016	1.466 (1.116–1.926)	0.006	1.311 (0.989–1.738)	0.059
Surgery+chemotherapy	1.443 (1.213–1.716)	< 0.001	1.231 (1.028–1.473)	0.023	1.430 (1.196–1.709)	< 0.001	1.233 (1.025–1.484)	0.026
BMI^c^								
Pathologic TNM stage								
Stage I	2.082 (1.130–3.839)	0.019	1.950 (1.037–3.666)	0.038	2.309 (1.183–4.504)	0.014	2.275 (1.155–4.482)	0.017
Stage II	1.693 (1.152–2.487)	0.007	1.811 (1.209–2.713)	0.004	1.743 (1.155–2.632)	0.008	1.859 (1.206–2.864)	0.005
Stage III	1.641 (1.357–1.986)	< 0.001	1.481 (1.215–1.806)	< 0.001	1.702 (1.399–2.070)	< 0.001	1.560 (1.272–1.913)	< 0.001
Age at diagnosis, years								
≥65	1.699 (1.307–2.210)	< 0.001	2.029 (1.537–2.677)	< 0.001	1.785 (1.342–2.373)	< 0.001	2.155 (1.594–2.914)	< 0.001
55 to <65	1.537 (1.163–2.032)	0.003	1.344 (1.007–1.794)	0.045	1.708 (1.289–2.263)	< 0.001	1.535 (1.147–2.054)	0.004
<55	1.753 (1.265–2.430)	0.001	1.376 (0.973–1.946)	0.071	1.736 (1.243–2.424)	0.001	1.314 (0.923–1.871)	0.130
Treatment arm								
Surgery only	1.762 (1.349–2.302)	< 0.001	1.490 (1.131–1.962)	0.005	1.899 (1.420–2.541)	< 0.001	1.611 (1.195–2.172)	0.002
Surgery+chemotherapy	1.691 (1.372–2.084)	< 0.001	1.684 (1.352–2.097)	< 0.001	1.758 (1.422–2.173)	< 0.001	1.697 (1.359–2.120)	< 0.001
NLR and BMI^d^								
Pathologic TNM stage								
Stage I	4.972 (1.525–16.218)	0.008	5.419 (1.453–20.204)	0.012	1.683 (0.229–12.358)	0.609	1.998 (0.244–16.361)	0.519
Stage II	2.651 (1.512–4.647)	0.001	3.341 (1.826–6.112)	< 0.001	2.827 (1.573–5.079)	0.001	3.624 (1.912–6.869)	< 0.001
Stage III	2.385 (1.750–3.250)	< 0.001	2.189 (1.580–3.032)	< 0.001	2.387 (1.738–3.278)	< 0.001	2.183 (1.564–3.047)	< 0.001
Age at diagnosis, years								
≥65	2.638 (1.752–3.973)	< 0.001	2.884 (1.836–4.529)	< 0.001	2.703 (1.746–4.183)	< 0.001	3.177 (1.950–5.178)	< 0.001
55 to <65	2.716 (1.692–4.359)	< 0.001	2.257 (1.358–3.750)	0.002	2.744 (1.687–4.463)	< 0.001	2.270 (1.347–3.824)	0.002
<55	2.773 (1.662–4.628)	< 0.001	2.381 (1.369–4.141)	0.002	2.573 (1.517–4.365)	< 0.001	2.066 (1.170–3.645)	0.012
Treatment arm								
Surgery only	3.554 (2.274–5.555)	< 0.001	3.016 (1.837–4.949)	< 0.001	3.463 (2.112–5.678)	< 0.001	3.012 (1.752–5.176)	< 0.001
Surgery + chemotherapy	2.599 (1.873–3.607)	< 0.001	2.481 (1.754–3.511)	< 0.001	2.579 (1.85–3.595)	< 0.001	2.425 (1.706–3.449)	< 0.001

^a^Cox proportional hazards models adjusted for age, sex, adjuvant chemotherapy, primary tumor location, histopathological differentiation, number of lymph nodes dissected, TNM stage, family history of gastric cancer, Charlson comorbidity score, smoke, alcohol, anemia, hypoalbuminemia, type of gastrectomy and surgical complications, unless stratified by those variables; ^b^All HRs compared patients with NLR ≥ 3 *vs.* patients with NLR < 3; ^c^All HRs compared patients with underweight (BMI < 18.5 kg/m^2^) *vs.* patients with BMI ≥ 18.5 kg/m^2^; ^d^All HRs compared patients with NLR ≥ 3 and underweight *vs.* patients with NLR < 3 and BMI ≥ 18.5 kg/m^2^. NLR, neutrophil-to-lymphocyte ratio; BMI, body mass index; HR, hazard ratio; CI, confidence intervals; TNM, tumor-node-metastasis.

Subgroup analyses showed that pathological TNM stage, age, and adjuvant chemotherapy had significant effects on the associations of NLR with survival. Compared with patients with low NLR, patients with high NLR had higher mortality in several subgroups (**Table 3**). In the subgroup analysis of pathological stage, NLR was not associated with survival in stage I patients using both univariate and multivariate analyses. In stage II patients, NLR was associated with prognoses using univariate analyses, but only exhibited a trend towards lower survival in the multivariable model. The effect of NLR ≥ 3 became significant in stage III patients, and the NLR ≥ 3 group had a worse OS (HR: 1.186, 95% CI: 1.003–1.404). In the subgroup analysis of age stratified by cut-off points at 55 and 65 years, NLR was not associated with survival in the young (< 55 years) or old patient (≥ 65 years) subgroups. In the ≥ 55 to < 65 years subgroup, the HRs for OS (HR: 1.485, 95% CI: 1.175–1.876) and RFS (HR: 1.422, 95% CI: 1.113–1.816) in the NLR ≥ 3 group were significantly higher than those in the low group. In addition, the subgroup analysis according to different treatments showed that elevated NLR predicted poor prognoses in both subgroups.

Using univariate analysis, there was no significant evidence that pathological TNM stage, age, or adjuvant chemotherapy changed the associations of BMI with OS or RFS (**Table 3**). In the subgroup analysis of pathological stage, worse RFS was observed in all of the underweight groups, including stage I (HR: 2.275, 95% CI: 1.155–4.482), stage II (HR: 1.859, 95% CI: 1.206–2.864), and stage III subgroup patients (HR: 1.560, 95% CI: 1.272–1.913). As the stage increased, the effect of BMI on mortality decreased. Similar results were observed for OS. However, in the subgroup analysis of age, BMI was not associated with survival in the young patient (< 55 years) subgroup, using multivariate analysis. In the ≥ 55 to < 65 years subgroup, the underweight group had a worse OS (HR: 1.344, 95% CI: 1.007–1.794) and RFS (HR: 1.535, 95% CI: 1.147–2.054). Likewise, in the ≥ 65 years subgroup, the underweight group had a worse OS (HR: 2.029, 95% CI: 1.537–2.677) and RFS (HR: 2.155, 95% CI: 1.594–2.914). Therefore, the importance of BMI on prognosis increased with age. In the subgroup analysis of treatments, adjuvant chemotherapy had a limited effect on the association of BMI with survival. Regarding adjuvant chemotherapy, underweight patients had a worse OS and RFS, and the HR of underweight patients in the adjuvant chemotherapy subgroup was similar to that of those in the surgery only subgroup.

In the entire group, patients with both NLR ≥ 3 and underweight had more than a 2-fold increased risk of death or relapse than patients with neither condition. The risk for OS was not changed by the stratification of stage, age, or adjuvant therapy (**Table 3**). Even stage I (HR: 5.419, 95% CI: 1.453–20.204), young (< 55 years) (HR: 2.381, 95% CI: 1.369–4.141), and adjuvant chemotherapy patients (HR: 2.481, 95% CI: 1.754–3.511) with both elevated NLR and underweight still had more than a 2-fold increased risk of death from any cause than patients of similar stage, age, and treatment but with neither condition. The HRs for RFS observed in subgroups according to age or different treatments were similar to those in the analysis of the entire group. However, the risk for RFS was modified by the stratification of the pathological stage. In early stage disease, elevated NLR combined with underweight did have a significant effect on RFS.

## Discussion

Prognostic biomarkers are crucial for the risk stratification of patients with gastric cancer and the selection of individualized treatment strategies after surgery. To the best of our knowledge, this is the first study to investigate the relationships between mortality and preoperative NLR and BMI in a large uniform cohort of patients who had undergone radical gastrectomy due to gastric or gastroesophageal junction adenocarcinomas. We found that elevated NLR or being underweight was associated with numerous unfavorable prognostic variables. Elevated NLR or decreased BMI at baseline had an independent prognostic effect on survival. Of note, we found that the co-occurrence of high NLR and being underweight (*vs.* neither) was associated with more than twice the mortality risk after surgery.

Although NLR and BMI have previously been shown to be associated with the prognosis in gastric cancer^12,16,17,25^ and many other solid tumors^11,19,20,22,26–30^, most studies have investigated them individually and in metastatic patients. In the present study, our results confirmed that similar processes occurred in resectable gastric cancer. In addition, the combination of NLR and BMI exerted a more potent prognostic effect than each index alone. Markers of systemic inflammation and nutritional status have an association with tumor-related characteristics^17,26,31–33^. In the present study, we further confirmed that the high NLR and underweight patients (*vs.* neither) had more aggressive tumors, as evidenced by increasing lymph node metastasis and advanced TNM stages. Underweight combined with inflammation nearly doubled the risk of anemia and hypoalbuminemia. The explanation could be that low BMI patients usually have poor nutritional reserves, and they were prone to develop malnutrition when complicated with high systemic inflammation. One study showed that high inflammation in the 24 months prior to diagnosis was associated with at-diagnosis sarcopenia and decreased BMI^34^. Furthermore, patients with both NLR ≥ 3 and underweight were in the high proportion of distal gastric cancer, and they were more likely to become combined with malignant pyloric obstruction and further aggravated malnutrition. However, we did not find a significant correlation between the at-diagnosis NLR and BMI using Spearman’s correlation analyses. A high systemic inflammation index did not indicate low BMI, and vice versa, so it is easy to understand that the combined use of NLR and BMI provides powerful prognostic information for risk stratification. The finding that the co-occurrence of high inflammation and being underweight was associated with poor prognosis is consistent with the limited prior studies in metastatic diseases. A study conducted in 313 patients with metastatic renal cell carcinoma treated with nivolumab showed that lower BMI combined with a higher inflammatory index tripled the risk of death^20^. In addition, we observed similar results independent of age, pathological stage, and postoperative chemotherapy in subgroup analyses.

Similar to the findings of previous studies^12,33^, high preoperative NLR was independently associated with poor OS and RFS in the entire group; however, little is known about the influences of NLR on postoperative survival in subgroup analyses. To the best of our knowledge, this is the largest study to examine the associations between NLR and survival in gastric cancer using subgroup analyses. In the subgroup analysis of pathological stage, NLR was not associated with survival in early stage patients, especially for RFS. In contrast with our findings, one previous study showed that elevated pretreatment derived NLR (dNLR) was an independent prognostic factor for OS in stage I gastric cancer patients^35^. The difference may be due to the use of dNLR, which is defined as the ratio of the neutrophil count to the white cell count minus the neutrophil count, instead of the NLR. Furthermore, the cut-off values were not the same. The subgroup analysis according to different treatments showed that elevated NLR predicted poor prognoses both in patients with adjuvant chemotherapy and in patients with surgery only. Our study is inconsistent with a meta-analysis in patients with prostate cancer that reported that elevated NLR predicted poor prognoses only in patients with adjuvant chemotherapy, but not in patients with surgery only^36^. The reason may be because most of the studies were not adjusted for postoperative complications in multivariable-adjusted models. Without adjusting for postoperative complications as one of the confounding factors, we observed similar results (data not shown). Considering the complexities between NLR and prognosis in subgroup analysis, the composition of patients may explain why most studies supported an association,^12,33,37^ but some studies failed to establish a link^38,39^. The exact mechanism of the association between high NLR and poor survival is still not clearly understood, but it may be due to the association of NLR with chronic inflammation and immune status^11,40,41^. The systemic inflammatory response can result in relative neutrophilia and lymphocytopenia^42^. Furthermore, a previous study showed that neutrophils can contribute to immune suppression and promote tumor progression^43^.

BMI has previously been shown to be a robust biomarker for outcomes in gastric cancer, including patients with resectable or metastatic disease^17,18,44,45^. In our study, in the subgroup analyses according to pathological stage, age, or adjuvant therapy, being underweight still correlated with both lower OS and RFS, except in patients < 55 years. The baseline BMI is usually low in healthy young people, meaning they are more likely to be underweight when diagnosed with gastric cancer but not underweight because of gastric cancer. The ability of the body to compensate for nutrition deficiency decreases with age. These facts may partially explain why the prognoses of younger patients were less likely to be affected by BMI than those of older patients. Similar results were observed in another retrospective large cohort study in patients with gastric cancer^17^. The prevalence rate of being underweight is relatively low in European and American patients with cancer^34^, but the incidence rate of gastric cancer is highest in Eastern Asia^1^. Among the patients in this study, 14% had a BMI less than 18.5 kg/m^2^ and 27% had a BMI less than 20.0 kg/m^2^ (data not shown), so being underweight was common. The prevalence rate of being underweight was higher than that reported by another study (4%) in Japanese patients^17^. The difference may be due to the high proportion of patients with advanced gastric cancer who are more prone to being underweight in our study. Our findings emphasized the importance of nutrition in patients with gastric cancer. Therefore, for patients with gastric cancer, in addition to conventional treatment, it is necessary to provide adequate nutritional support, especially for underweight patients.

As in any observational study, several potential biases should be considered. First, due to the cross-sectional nature of this study and the lack of temporal order in our data, we could not separate the causality between systematic inflammation and being underweight. Second, the detailed postoperative treatments were beyond our control. However, we investigated the associations in the subset according to adjuvant chemotherapy. Furthermore, it should be recognized that NLR is a nonspecific parameter, and it could be influenced by concomitant diseases, such as infection, autoimmune disease, or stroke^11,46,47^.

## Conclusions

Both decreased BMI and elevated NLR were independent prognostic biomarkers in resectable gastric cancer. We also found that a low BMI combined with a high systemic inflammation index identified patients with more than a 2-fold risk of mortality compared to patients with neither condition. Considering that underweight and high NLR are common in gastric cancer patients, further prospective studies are needed to confirm our findings and verify whether increasing the BMI or suppressing inflammation can enhance survival.
