# Supplementary material for: Association of systemic inflammation and body mass index with survival in patients with resectable gastric or gastroesophageal junction adenocarcinomas
Source: Cancer Biol Med. 2021 Feb 15;18(1):283–97. doi: 10.20892/j.issn.2095-3941.2020.0246 (PMC7877168; doi:10.20892/j.issn.2095-3941.2020.0246)
Supplement: Supplementary file 1 [file cbm-18-283-s001.pdf]

Supplementary materials

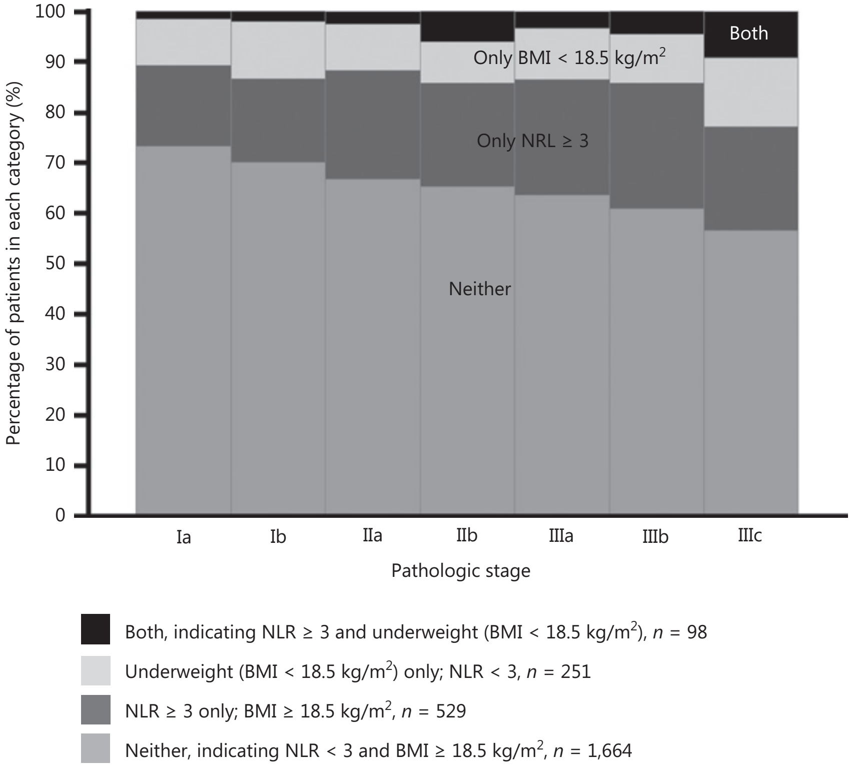

Figure S1 Pathological stage distribution according to neutrophil-to-lymphocyte ratio (NLR) and body mass index (BMI) at diagnosis in patients with resectable gastric or gastroesophageal junction adenocarcinomas.

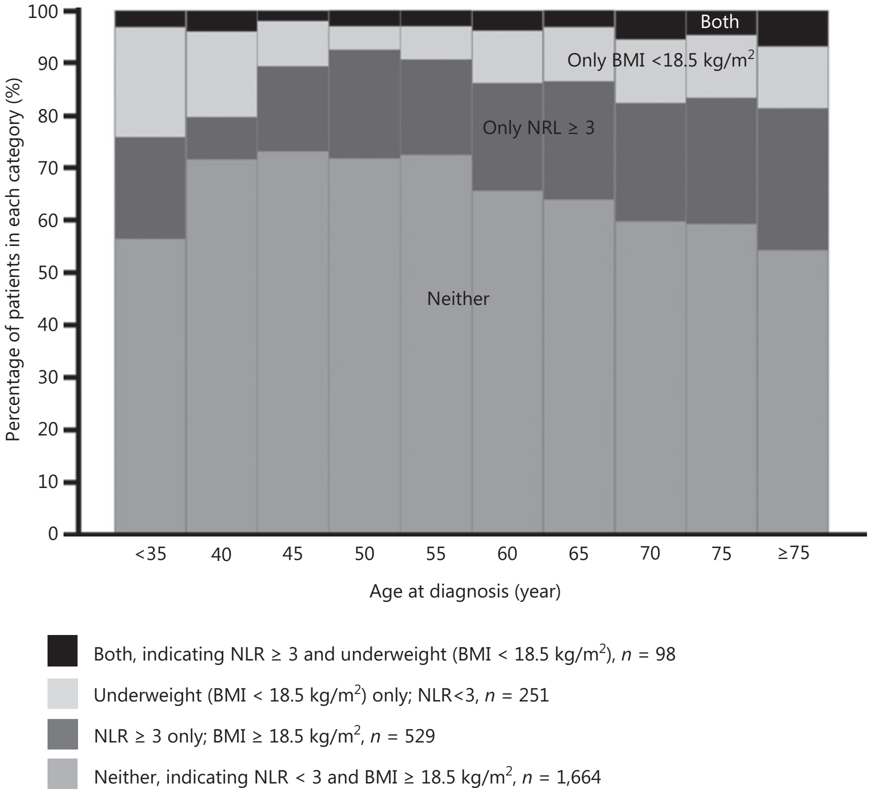

Figure S2 Age distribution according to neutrophil-to-lymphocyte ratio (NLR) and body mass index (BMI) at diagnosis in patients with resectable gastric or gastroesophageal junction adenocarcinomas.
